# Adaptive and Economically-Robust Group Selling of Spectrum Slots for Cognitive Radio-Based Networks

**DOI:** 10.3390/s18082490

**Published:** 2018-08-01

**Authors:** Maqbool Ahmad, Muhammad Shafiq, Azeem Irshad, Muhammad Khalil Afzal, Dae Wan Kim, Jin-Ghoo Choi

**Affiliations:** 1 Department of Digital Convergence Business, Yeungnam University, Gyeongsan 38541, Korea; maqbool.pu@gmail.com; 2 Department of Information Technology, University of Gujrat, Gujrat 50700, Pakistan; 3 Department of Computer Science & Software Engineering, International Islamic University, Islamabad 44000, Pakistan; irshadazeem2@gmail.com; 4 Department of Computer Science, COMSATS Institute of Information Technology, Wah Cantt 47040, Pakistan; khalilafzal@ciitwah.edu.pk; 5 Department of Business Administration, Yeungnam University, Gyeongsan 38541, Korea; c.kim@ynu.ac.kr; 6 Department of Information and Communication Engineering, Yeungnam University, Gyeongsan 38541, Korea

**Keywords:** cognitive radio network, dynamic spectrum access, group-buying, group-selling, IoT, spectrum auction

## Abstract

Auction theory has found vital application in cognitive radio to relieve spectrum scarcity by redistributing idle channels to those who value them most. However, countries have been slow to introduce spectrum auctions in the secondary market. This could be in part because a number of substantial conflicts could emerge for leasing the spectrum at the micro level. These representative conflicts include the lack of legislation, interference management, setting a reasonable price, etc. In addition, the heterogeneous nature of the spectrum precludes the true evaluation of non-identical channels. The information abstracted from the initial activity in terms of price paid for specific channels may not be a useful indicator for the valuation of another channel. Therefore, auction mechanisms to efficiently redistribute idle channels in the secondary market are of vital interest. In this paper, we first investigate such leading conflicts and then propose a novel Adaptive and Economically-Robust spectrum slot Group-selling scheme (AERG), for cognitive radio-based networks such as IoT, 5G and LTE-Advanced. This scheme enables group-selling behavior among the primary users to collectively sell their uplink slots that are individually not attractive to the buyers due to the auction overhead. AERG is based on two single-round sealed-bid reverse-auction mechanisms accomplished in three phases. In the first phase, participants adapt asks and bids to fairly evaluate uplink slots considering the dynamics of spectrum trading such as space and time. In the second phase, an inner-auction in each primary network is conducted to collect asks on group slots, and then, an outer-auction is held between primary and secondary networks. In the third phase, the winning primary network declares the winners of the inner-auction that can evenly share the revenue of the slots. Simulation results and logical proofs verify that AERG satisfies economic properties such as budget balance, truthfulness and individual rationality and improves the utilities of the participants.

## 1. Introduction

In the last decade, the Internet-of-Things (IoT) [1] has received much attention due to the low-price and low-power smart devices like sensors, actuators, Radio Frequency ID (RFID) tags, etc., that can connect to the Internet anytime, anywhere for anything. Despite its virtues, IoT mostly relies on license-free Industrial-Scientific-and-Medical (ISM) bands using wireless technologies such as WiFi, ZigBee, LTE-Advanced, 6LowPAN, M2M, RFID, etc., to connect the tremendous number of devices to the Internet. It is expected that by 2025, Internet-connected devices will be 10 times more than the human population [2]. IoT devices are, therefore, anticipated to demand a huge amount of wireless spectrum in the near future, but even now, the available frequency band is not enough. Moreover, the proliferation of Internet-connected devices can make the ISM bands unavailable due to the anticipated spectrum shortage and high congestion. To resolve this spectrum shortage issue, Cognitive Radio (CR) technology can be considered as one of the possible solutions [3]. The CR system can let Secondary Users (SUs) enable dynamic spectrum access so that they freely operate across the licensed and unlicensed spectrum bands [4], while the traditional wireless radio can only access unlicensed ISM-bands-based networks like Bluetooth, IEEE 802.11, HiperLAN, and so on. The SUs not only access the unlicensed bands, but they can coexist with the Primary Users (PUs) in different primary networks such as GSM, GPRS, UMTS and TV bands. Therefore, the CR system has great potential to promote efficient use of the spectrum that is otherwise misused by the licensed users.

The PUs in the CR system should, however, address the key issue of how to release their allocated spectrum bands to the SUs first. Towards this end, reselling of PUs’ free slots in the secondary market looks purposeful due to the following two facts. First, the PUs already have ample idle slots in totality in their allocated frequencies to accommodate the activities of SUs [5,6,7]. Second, the newly-freed frequencies in the primary market cannot be afforded by SUs due to very high prices [8,9]. However, PUs cannot be enforced to accept the transmission activities of SUs because the PUs have acquired the exclusive right on the channel legitimately. The PUs are not supposed to voluntarily release the spectrum channels for SUs. Therefore, we need incentives for PUs. The auction mechanism is an efficient way to motivate the PUs to share their licensed channels with SUs. By introducing the auction-based secondary markets, the PUs will lease their uplink slots in licensed channels to SUs when the channels are not used temporarily, and the SUs will purchase the slots whenever they are necessary. The PUs earn extra profits without sacrificing their communication quality, which becomes a strong incentive to share the channels with SUs. The SUs enjoy the higher channel availability with reasonable cost. More importantly, utilization of the scarce spectrum channels increases, as well.

The auction-based trading model has been a widely-used concept in the selling and buying of the valuable goods that have an unpredictable and variable price, like the radio spectrum. The bidders (or buyers) usually report their bids to the auctioneer (or seller), which may consist of the price and some additional information, such as the quality of the traded goods. On the other hand, the seller opens the auction, receives the bids and decides the price and winner of the auction based on a particular algorithm of choice. There four basic types of auction algorithm (or simply the auctions) that are extensively envisioned and analyzed in industry and academia [10,11,12], as follows.

### 1.1. Ascending-Bid Auction

**Figure 1:**
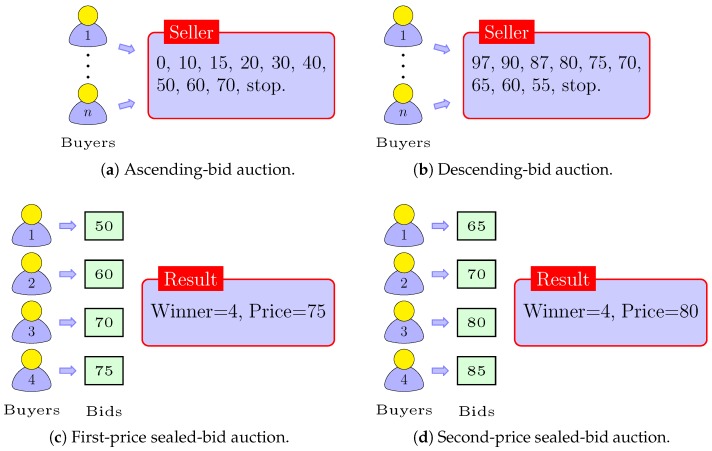
Illustrations of the various types of auctions.

Figure 1a illustrates the operation of the ascending-bid auction scheme, which is also known as the English auction. In this type of auction, the bidders usually interact with the auctioneer in real time when a single unit is being sold. The auctioneer successively raises the price, while each bidder necessarily calls out the bid value of the item such that no other bidders have yet called out. Therefore, the bidders drop out gradually, until only one bidder is left that wins the item at the final (or stepped) price. This type of auction usually reveals the bid information of the runner-up bidder to define the final bid. The most popular forms of the ascending-bid auctions are oral auctions, in which bidders call out their bids either physically or electronically.

### 1.2. Descending-Bid Auction

The operation of the descending-bid auction is done exactly opposite that of the ascending-bid auction. We illustrate the descending-bid auction in Figure 1b, in which the auctioneer first starts with the very high price and then lowers the price gradually, while the willing bidders accept the price in an interactive format. Therefore, the bidder who will accept the final price at the first moment wins the item at that price. The descending-bid auction schemes are often called Dutch auctions because in The Netherlands, they are commonly used for the sale of flowers.

### 1.3. First-Price Sealed-Bid Auction

Figure 1c illustrates the first-price sealed-bid auction, in which the bidders simultaneously submit the sealed-bids to the auctioneer. The terminology of sealed-bids actually comes from the basic format of such auctions, in which the bids were submitted to the auctioneer in a sealed cover. When the auctioneer opens the sealed-bids of all the contestants, the bidder with the highest bid is declared as the winner of the item. Therefore, the winner is liable to pay the price equal to the value of its own bid. In this kind of auction, the bids of the winners are much less certain about the bids of the opponent bidders, unlike the ascending and descending-bid auctions. This effect, however, makes the first-price sealed-bid auctions less profitable compared to the ascending- and descending-bid auctions.

### 1.4. Second-Price Sealed-Bid Auction

The second-price sealed-bid auction is also known as the Vickrey auction in honor of Prof. William Vickrey, who first coined the model of this kind of auction scheme [13]. We illustrate the operation of the second-price sealed-bid auction in Figure 1d, in which the bidder with the highest bid wins the item and then pays the price equal to the value of the second-highest bid (i.e., the highest bid of the losers). The model of the second price sealed-bid has been very popular in economics because it can make the auction schemes truthful. The world’s largest online auction, namely eBay, is the famous example of second-price sealed-bid auction schemes.

In general, the auction framework also appears to be most suitable because it can capture the most salient features of the spectrum sharing problem. First, the auctioneer does not know the current value of the commodity in which the bidders are interested, which enables an auctioneer to get a good estimation of how much value it has to the bidders. Second, auctions can be effectively designed for the sake of efficient allocation of the commodities to the bidders who need and value them most. Third, auctions have less time delay due to the minimum interaction required between the auctioneer and the bidders. Fourth, auctions are highly volatile with respect to the demands of the commodity compared to the other pricing schemes. Fifth, auctions may also relax the undue price competition between the bidders rather than increasing the winners’ curse. Last, but not least, auctions place control in the hands of those that have direct concerns about defining the price and allocation strategy. All these aspects make the auction mechanism more realistic compared to the other market-based pricing mechanisms.

Auctions with a flawed design might have adverse impacts, which can introduce inefficiencies in the trading model. Hence, an auction scheme must satisfy the economic properties, as in [14,15]. An auction can be economically-robust if it is truthful, budget balanced, individually rational and system and usable efficient. An auction is truthful if no participant has an incentive to lie or collude, so that a selfish bidder cannot cheat or hurt the interest of the others. To make the auction budget balanced, the total gain of the auctioneer must be non-negative, which makes an auction self-sustained with the non-negative payoff. An auction is individually rational if no winning bidder is charged more than its bid submitted to the auctioneer, so that its interest remains intact. An auction is said to be system efficient if the competitive advantage (or the sum of valuations of all the bidders) is maximized. An auction is usable efficient if it permits channels to be reused by the participants that are far apart geographically in order to maximize the social welfare. However, there exists a tradeoff among some of these properties [16]. For example, an auction cannot be truthful and system efficient simultaneously since both features mutually exclude each other, ensuring one of them might come at the expense of the other.

We can witness related studies on the auction-based spectrum trading models. The conventional schemes cannot be directly applied to the spectrum trading models due to their economic inefficiencies and the peculiarities in the nature of traded goods [17]. The early studies on the spectrum auction designs were based on single seller and multi-buyer auction mechanisms [18,19]. Some studies bear the features of truthful auction design, as in [15,20]. Nonetheless, these schemes did not consider the secret dealings on the part of both auctioneers and bidders. Later on, THEMIS as suggested to focus on enabling security mechanism for the interests of the participant involved in the leasing process [21]. Some other truthful spectrum-auction schemes were proposed in [15,18]. However, these schemes grossly drop one group of bidders since the clearing price is taken as equivalent to the bid value of the group. Thereafter, the authors enhanced this trading model by sacrificing one buyer from every group, as in [22]. Further, an interference-graph based model was proposed for spectrum allocation in order to avoid interference and ensure spectrum reusability among the multiple users [18,23]. Then, MTSSA was put forwarded to ensure the spectrum reusability and truthfulness of the reported bids [24]. In this context, a more recent spectrum auction scheme based on the exponential-scale version was proposed in [25]. A similar auction-based scheme can be found in [26], which is based on the physical interference model by allowing multiple SUs to continue the leasing process of the same spectrum at the same time. We can follow some detailed studies on spectrum sharing schemes for cognitive radio-based IoT networks, 5G and next-generation cellular-networks in [27,28,29].

For the spectrum trading problem, we can also find different game theoretical models in literature, such as the Stackelberg game [30], non-cooperative game [31], potential game [32], and so on. Nevertheless, auction theory is the preferred method to solve the spectrum sharing problem, because it can sufficiently protect the economic incentives of all the participants [33,34]. In spectrum auctions, we can classify the state-of-the-art contributions into individual-buying, group-buying and group-selling schemes, as inscribed in Table 1. Among the individual-buying schemes, a certain system allows some SUs to create a group, and when the group wins the contest, the members can use the whole spectrum bands. Examples of famous individual-buying schemes are WERITAS [18], SMALL [35], TRUST [15], TASC [36], TAHES [37], SMSHER [38], and so on. However, this is very different from group-buying schemes. In group-buying schemes, a group collects the budgets of all the members to increase the bargaining power. With a big group budget, a group can purchase commodities that are not affordable individually. In [39], the authors first introduced the concept of group-buying and proposed TASG, which supports group-buying of SUs to compensate the limited budget and poor bargaining power of the individual user. We can find some more group-buying schemes like DEAL [40] and TRUB [41], but they are the straightforward extension of TASG. There is a much work that has tangled the spectrum reusability in the auction design to solve the spectrum scarcity problem, e.g., TRUST, SMALL, TASC, etc. However, no scheme either in individual-buying or group-buying auction exists that can enable group-selling behavior among the resource-constrained sellers. We can stop the leakage (or wastage) of uplink spectrum slots that are otherwise not leased (or reused) by giving them to the most urgent users in the secondary market. This is because of the fact that neither the buyers nor the sellers are motivated to buy and sell the fraction of spectrum in so small units at the micro level. If the uplink free slots are merged in a group at space and time granularity, the buyers and the sellers are easy to attract, such that sellers can earn more revenue and the buyers can get more spectrum. Therefore, the group-selling concept is very meaningful, especially in IoT, where we need more and more spectrum, day by day.

We have introduced AERG, which was inspired by TASG. To the best of our knowledge, AERG is the first scheme that supports group-selling of spectrum slots in the secondary market. In AERG, PUs collectively sell their uplink idle slots, which are otherwise individually wasted in the network due to the less market value. That is, the resource-constrained PUs at one time and in one space assemble their uplink slots in a group to compete in the auction for the higher revenue by enabling group-selling behavior among them and raise the market value of their offered slots. If the created group sells the group slots successfully, the involved PUs can share their realized revenue together. In return, PUs are not only encouraged to release the idle slots, but the buyers are also motivated to get more spectrum. In AERG, the participants are further allowed to adapt their personal asks/bids to improve the social welfare and spectrum efficiency. On the contrary, we can find some individual-selling schemes such as [42], in which PUs can individually sell their spectrum slots. However, buyers are not motivated to purchase the free slots one by one from each seller. In that case, auction overhead can be increased from the cost of the individual slots. Therefore, the slots’ group-selling is of interest, since PUs do not have many idle slots due to their intermittent needs for the Internet and limited resources.

The key contributions of this paper are summarized as:

- We explore the key issues of spectrum trading with online auctions that make the implementation of the dynamic access strategy difficult in the secondary market. Using examples from a range of countries, we discuss the ways in which governments and individuals try to deal with the leading issues and highlight their causes and effects on the spectrum trading in the world today.
- We first develop a group selling framework of spectrum slots for cognitive radio-based Internet-of-Things. The design of proposed framework is based on the two nested auction algorithms, which is aimed at enabling PUs to sell their uplink slots in the group that otherwise wasted them in the network since they are individually not attractive to the SUs due to auction overhead.
- We develop the slots’ evaluation algorithms that adapt their asks and bid prices considering the dynamics of spectrum trading. The underlying objective is to improve the utilities of the participants such that sellers are motivated to release more and more slots and buyers are encouraged to get more spectrum in return.

The rest of this paper is organized as follow. Section 2 gives an overview of spectrum leasing’s key issues in the secondary market. Section 3 describes the system model. Section 4 presents the proposed auction scheme. Section 5 proves the economic properties of the proposed auction, and then, its performance is evaluated in Section 6. Finally, in the last section, we conclude the paper.

## 2. Issues of Spectrum Leasing in Secondary Markets

Despite the compulsive need for radio spectrums, most countries are slow to adopt the dynamic spectrum access strategy. In this section, we discuss the issues that should be resolved for the rapid spread of this new spectrum paradigm.

### 2.1. Legislation and Standardization

Spectrum leasing by auctions is not popular yet, even in the primary markets. There are only a handful of countries that adopt the auction, e.g., the United States, Canada, Australia, New Zealand, Korea, Guatemala, Germany and Pakistan. The rest of the countries still manage the spectrum resources through the traditional command and control [43]. The countries with auction-based primary markets have endeavored to develop the secondary markets [44]. However, little attention has been paid to establish regulations for secondary markets. To invigorate the market, it is imperative to define the rights and obligations of the involved parties. The national and international regulators should cooperate to deal with the issues arising from the lack of legislation and standardization. Technology proliferates when legislation and standards come along. In this sense, the recent IEEE 802.22 standard can be a cornerstone, which enables the sharing of unused television bands with the unlicensed users in un-served or under-served rural communities [45]. The motivation for this standard has come from the fact that all TV stations are not active all the time, so the spectrum bands can be exploited to gain additional benefits when idle. However, in the standard, the main concern is focused on protecting the television operators from the opportunistic SUs. We need more complete legislation and standards to promote the spectrum leasing activity in the secondary markets.

### 2.2. Flexible Service Migration

A spectrum license is contracted for a specific service for long-term periods, and it can be extended if the license holder abides by the contract faithfully. Once a service provider acquires the license, it is not replaced by others to introduce a new service, even if the spectrum is not utilized at all. The static allocation policy provides a strong security to spectrum license holders, which encourages the investment in the infrastructure. However, this sink cost hinders the existing service providers, which migrate to new services. Moreover, current regulations do not allow the license holders to change the service before the contract period ends. For example, in the television service of the United States, at least 8 MHz of spectrum for each band is not being utilized, but the channels cannot be exploited for other services [46]. To improve the spectrum efficiency, regulations should be flexible for the existing service providers to return the spectrum license before the contract period ends, and to migrate to new services the customers require.

### 2.3. Reserve Price

In auctions, a seller can set a reserve price on the commodity, which is the minimum price placed by the auctioneer internally. It plays an important role especially when the seller does not know the value of commodities. The reserve price is not mandatory, but an auction held in New Zealand advocates the necessity of the reserve price. In 1990, a spectrum auction was conducted in New Zealand without the reserve price. The result was disastrous. The highest bidder bid $100,000, but it won the spectrum license just for $6. In another case in point, the winning bidder bid $7,000,000, but it won the spectrum license only for $5000 [47]. Actually, the reserve price is a double-edged sword. It can increase the revenue of the seller by guaranteeing the minimum profit once the commodity is sold. However, the reserve price discourages the potential buyers from participating in the auction when their budgets are limited. When the competition is mild, the commodity may not be sold, and the seller’s revenue decreases [48,49].

**Figure 2:**
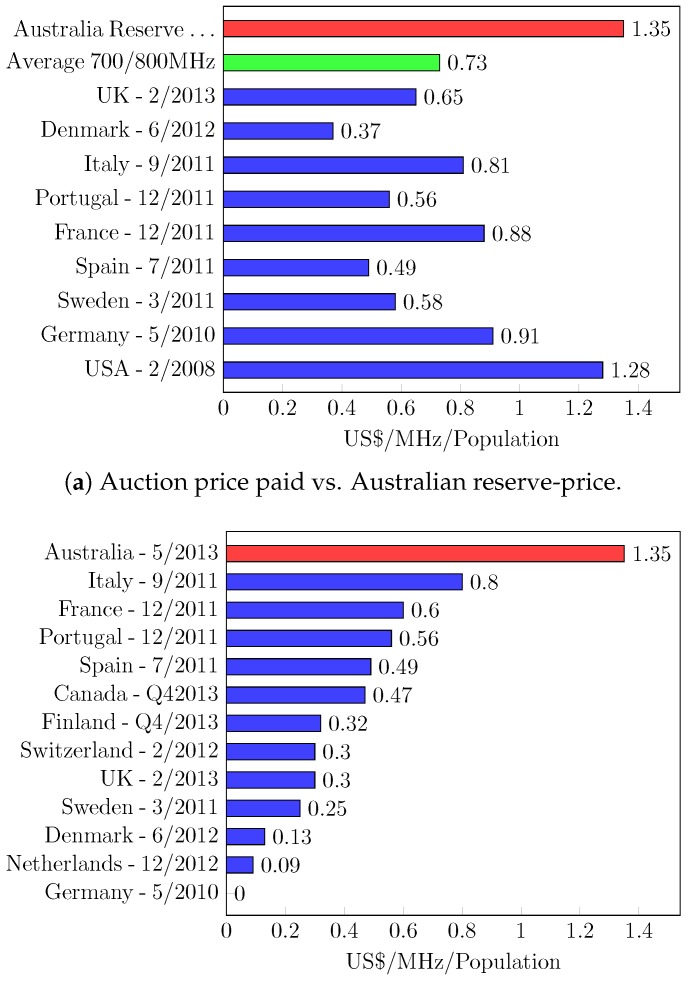
The reserve price analysis of the 700/800 MHz spectrum auctions held in various countries.

As the effects of the reserve price are two-fold, we should set it at a proper value. The extreme values can negatively affect the seller’s revenue. Let us see an example of the auctions with a too high reserve price. In 2013, a spectrum auction for the 700-MHz band was held in Australia [50]. The reserve price therein was set to $1.35/MHz/population, which was 186% higher than the average price of the same portion of spectrum (see Figure 2a). As a result, 30 MHz out of the 90-MHz spectrum had been unsold, and the Federal Government of Australia is estimated to have had a 100% loss for freeing up the nationwide block of radio spectrum. Oppositely, we also have an example of the auctions with too low a reserve price. A spectrum auction, conducted in Germany in 2010 [51], raised $5.5 billion of revenue, which was much lower than expected. As shown in Figure 2b, the reserve price was set to one cent/MHz/population [52,53]. This is very low considering the economy of German. The estimates by KPMG (i.e., one of the world’s leading firms of auditors) took everyone by surprise that the German auction could have raised between $7.5–10 billion revenue [54], but its improper reserve price has left almost $2–4.5 billion on the table.

### 2.4. Trustworthy Framework

In the traditional spectrum allocation, the involved parties know each other well and trust is easily ensured. On the other hand, the situation is totally different in the secondary markets since the spectrum leasing is usually processed online. In online trades, a trustworthy framework is essential to prevent and detect fraud, as a player cannot ensure the true identity of the counterpart. Even worse, spectrum deals can be performed through the vulnerable wireless medium. In the literature, we can find two candidate trust management schemes: reputation-based schemes and policy-based schemes [55]. Unfortunately, both schemes assume that the trust is independent of time. Therefore, they do not match our case since the faithfulness of the market players can change over time [56]. Especially, sellers can set a low reserve price intentionally to pay fewer fees to the regulator or pretend to be a legitimate bidder to push up the sale price [57]. Without a trustworthy framework, we cannot detect the anomalies, and the buyers are liable to lose time and money.

### 2.5. Payment Mechanism

When the players are exposed to financial risks such as fraud and credit deception, the market becomes unstable. Therefore, for the sustainable operation of secondary markets, we need a secure and efficient payment mechanism. We can classify the payment methods into prepaid schemes and postpaid schemes. In the prepaid schemes, buyers should pay before using the spectrum while they pay for the spectrum after using it in postpaid schemes. The prepaid schemes are safer for sellers, but the schemes are not adequate for auction-based markets. The postpaid schemes are advantageous for buyers and flexible. Therefore, we prefer a postpaid style payment scheme for the secondary markets. Along with that, we need to compensate the postpaid payment schemes to alleviate the financial risk of sellers. In Ref. [58], a seller’s concerns about a buyer’s credit have been addressed. Thereby, the seller determines the reputation of buyers by the recommendations of external authorities and its personal experiences. Similarly, a buyer has concerns about the seller’s credit. In Ref. [59], each buyer chose a seller based on the seller’s reputation, service duration and spectrum parameters such as bandwidth and signal quality. However, these approaches do not eliminate the financial risk of both parties since the reputation or credit changes over time. We need a payment scheme that incorporates the temporal factors, as well as the recommendations and personal experiences.

### 2.6. Perceived Risks

Users in an open market naturally have greater risk of uncertain demand that essentially impacts the determination of actual prices and investment decisions in a newly-liberalized sector such as spectrum leasing in the secondary market. This may reduce the activity of market participants and thus likely lessen the information of spectrum trading. Simply put, the ability to lease spectrum in a secondary market without the fear of underselling or overpaying is, in fact, bounded by a number of reasons [60]. First, information may be scarce in that secondary market in which, unless given time to learn and mature, participants cannot be confident about the real value of the spectrum. Second, the heterogeneous nature of the spectrum precludes a true valuation of non-identical frequency bands. Therefore, information abstracted from the initial activity in terms of price paid for a specific frequency band may not be a useful indicator for the valuation of another frequency band. Third, the perceived risk of launching new services by the operators ultimately increases due to the huge sink costs required for developing network infrastructure despite getting these services functionally over the spectrum bands obtained at high prices.

For example, the first auction that Pakistan carried out in 2014 [61] for 3G/4G mobile services yielded disappointing results. Four out of the five telecom companies submitted their bids; four licenses of 3G, and only one license of 4G was bought. No new company showed interest, nor did the two existing foreign companies (i.e., Turkcell, Saudi Telecom) enter into the contest. The auction revenue raised over just $1.1 billion compared that of the $4–5 billion estimated results by the PTA (Pakistan Telecommunication Authority). More interestingly, a petition was also submitted by a political party (Watan) to the country’s Supreme Court about undervaluation of the spectrum being sold. This dilemma is particularly evident for the secondary market, as well, which can essentially impose perceived risks on market players to effectively learn and control the uncertain behavior of spectrum trading. Given the unpredictability and perceived risks, stakeholders may make the wrong decisions while making judgments about the valuation of the radio spectrum.

### 2.7. Market Information Management

Players of a market require a variety of information. Actually, supply and demand are the most important information for the efficient participation in the trades. However, others can be beneficial depending on the situation. Especially, in CR-based markets, a primary network can provide the spectrum information to secondary networks such as time of availability, history prices, service quality, bandwidth, spectrum policy, contract duration, etc. While managing and providing the information in public, we can improve the transparency of markets and promote the spectrum efficiency by building the confidence in involved agents. In the United States, the FCC has recently ruled in favor of information sharing for the opportunistic access of TV broadcast bands [62]. However, many of the details about the infrastructure of market information and the access model are still left undecided.

## 3. System Model

**Figure 3:**
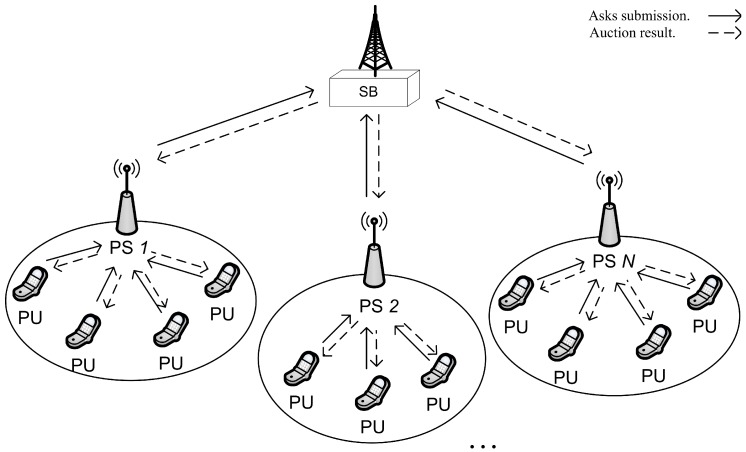
System model. SB, Secondary Buyer; PS, Primary Seller; PU, Primary User.

We now briefly discuss the system model of the proposed auction framework. For simplicity, we have considered *N* primary networks and one secondary network in a CR network, as shown in Figure 3. An intermediate agent in each primary network delivers the services of Primary Seller (PS) and the service access point of the secondary network plays the role of Secondary Buyer (SB) and renders services to multiple SUs. There exists $N_{i}$ number of PUs in PS *j* that sell their uplink idle slots to the SB through the reverse-auction. In general, our proposed system is based on the two-stage reverse-auction mechanism, wherein the outer-auction is held between PSs and SB and the inner-auction is carried out between each PS and its serving PUs. The ultimate buyers of the slots are SUs, but each SB takes part in the outer-auction on behalf of the SUs. We assume that there exists infrastructure such as a central bank and control channels that enables the exchange of money and information between the entities involved in the auction.

**Figure 4:**
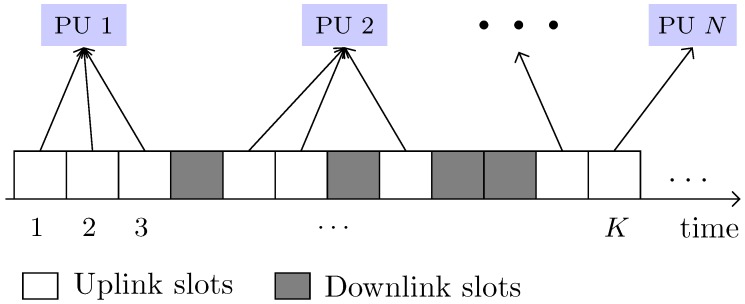
Channel view of the system model.

In the proposed system, we have assumed *n* consecutive periods with an identical length. At the beginning of each period, PUs sell their uplink idle slots to harvest revenue, and SB purchases those slots for communication. As illustrated in Figure 4, PS *i* has *K* slots in a channel *c*. Among those PU, *j* can only release $k_{ij}^{c}$ uplink slots to the secondary market. On the contrary, SB instead of dealing with multiple PUs (for the very limited number of spectrum slots) would like to get more and more spectrum from a single seller to reduce overhead. Given this limitation, multiple PUs in each PS can jointly offer their group of slots $k_{i}^{c}$ by group-selling to increase their revenue and bargaining power. The characteristics of slots in a channel vary according to time and space granularity, in terms of availability, signal-to-noise ratio (SNR), maximum allowed transmission power, and so on. Hence, the evaluation for slots can be different for PUs at each location and time.

For the efficient trade of spectrum slots, we have proposed a reverse-auction mechanism that consists of three phases called adaption phase, auction phase and remuneration phase. In the adaption phase, each PU *i* belonging to PS *j* first adapts the reserve-ask ${\tilde{A}}_{ij}^{c}$ as the maximum valuation on slots of channel *c* to define its ask $A_{ij}$. Similarly, each SB places the maximum price for $k_{i}^{c}$ slots, which is called the reserve-bid and is denoted as ${\tilde{B}}^{c}$. The SB adjust the reserve-bid dynamically considering the past information of asks to the spectrum slots. The values of reserve-ask and reserve-bid are not open to the public because both are private to the PU and SB, respectively.

In the auction phase, PU submits asks ${\left\{A_{ij}^{c}\right\}}_{c=1,\cdots ,C}$ to the serving PS for the inner-auction. Therein, PUs also announce time information on available slots with their ask submissions. In response, PS collects asks from PU *j* and computes one group ask $A_{i}^{c}$ for each channel *c*. Subsequently, the PS submits group asks ${\left\{A_{i}^{c}\right\}}_{c=1,\cdots ,C}$ to the PS on all the channels for the outer-auction. We assume that PSs also disseminate time information on slots to the SB with their group asks. The SB purchases the slots on channels considering asks from PSs. We denote the set of winners of the outer-auction as W, which contains the tuples of a winning PS and its purchased slots’ channel index. When PS *i* sells $k_{i}^{c}$ slots (or group-slots) successfully, we denote PS i∈W and/or group-slot $k_{i}^{c}\in W$ with some abuse of notation. After getting $k_{i}^{c}$ slots, SB *i* constructs a winner-set $W_{i}$ of PSs for the *k* slots of channel *c* and announces the result to its PSs. To clear the account, SB pays an amount of $P^{c}$ to the winning PS *i* of purchased slots for channel *c*, which becomes revenue of the PS. Later on, PS *i* also makes a payment of $P_{ij}^{c}$ to the PU $j\in W_{i}$ that becomes revenue of the PU. From channel *c*, we denote the revenue of PS *i* and PU *j* as $R_{i}^{c}$ and $R_{ij}^{c}$, respectively. A PS may pay the PUs less than what it should receive from the SB. However, the individual PU should not be paid less than its ask. The winning PU *j* of PS *i* has the gain of $R_{ij}/\left|W_{i}\right|$ by sharing the earned revenue of $k_{i}^{c}$ slots, where |A| denotes the cardinal number of set A. Thus, it has the utility of:

(1) $$U_{ij}=\left\{\begin{array}{cc} \frac{R_{ij}}{|W_{i}|}\times k_{i}^{c}, & \mathrm{if}\;\mathrm{PS}\;i\in W\;\mathrm{and}\;\mathrm{PU}\;j\in W_{i}\\ 0, & \mathrm{otherwise}. \end{array}\right.$$

An PS *i* receives the revenue of $R_{i}^{c}$ from the SB and pays $\sum_{j\in W_{i}}P_{i}$ to the PUs. Therefore, its utility is given as:

(2) $$U_{i}=\left\{\begin{array}{cc} R_{i}-\sum_{j\in W_{i}}P_{i}, & \mathrm{if}\;\mathrm{PS}\;i\in W\\ 0, & \mathrm{otherwise}. \end{array}\right.$$

We mention that if an PU or an PS cannot sell the slots, it has then zero utility. However, the utility of SB is the difference between its valuation of slots in terms of reserve-bids and payments to the PSs, which can be written as,

(3) $$U=\left\{\begin{array}{cc} {\tilde{B}}^{c}-\sum_{i\in W}P^{c}, & \mathrm{if}\;\mathrm{PS}\;i\in W\\ 0, & \mathrm{otherwise}. \end{array}\right.$$

In the remuneration phase, PS i∈W formally announces the result of inner-auction, and then, it will make a payment to PU $j\in W_{ij}$ exactly equal to the clearing price $k_{i}^{c}$ for each slot. Finally, we discuss another performance metric, social welfare *S*, to evaluate how efficiently spectrum slots are released into the system i.e.,

(4) $$S≜\sum_{i\in W,j\in W_{i}}A_{ij},$$

which is what is the sum of the valuations of all the winning sellers in terms of their ask values. We refer to the results of TASG [39] and SMASHER [38] for Equations (1)–(4), respectively.

Before proceeding, we summarize the representative symbols for quick reference in Table 2.

## 4. Proposed Auction Framework

We have reviewed the system of proposed auction called AERG (Adaptive and Economically-Robust Group selling of spectrum slots for group-users in the secondary market). Still, some important issues have not been addressed such as how SBs choose the winning SUs in the outer-auction, how SBs compute group ask from the asks of PUs for each group slot, how SB allocates channels to SUs in inner-auction, how SB adjusts purchased prices of the spectrum slots, etc. In this section, we elaborate on the AERG framework to address all these issues. In AERG, spectrum slots are leased to SUs for a certain time period. The SUs return the leased channel slots after the period, and the next period begins. At the beginning of each period, the auction is processed in three phases including the adaption phase, auction phase and remuneration phase as shown in Figure 3. We now describe each phase in detail.

### 4.1. Adaption Phase

There exists a conflict of interest in the spectrum market. Therein, the buyer always wants to purchase the channels at the minimum price, while the seller wants to get the maximum return. In spectrum auctions, SB essentially sets the reserve-bids according to the current value of the channel slots. If SB sets the reserve-bid less than the actual price, it cannot purchase the spectrum slots. In contrast, if the reserve-bid is too high, then it can cause substantial loss to the SB. On the other hand, if PUs set the asking price of the spectrum slots too high, spectrum slots are unlikely to be sold. Further, the lower asks will hold back its revenue. To this end, we have developed the following algorithms to appropriately adjust the spectrum valuations by the participants.

#### 4.1.1. Ask Adaption

We discuss how PUs can adapt their personal asks of spectrum slots offered to the secondary market. For period *n*, we denote the availability of slots (or supply) in channel *c* as $L_{ij}^{c}$. We model the reserve-ask as inversely proportional to the supply, i.e., $A_{i}=a\frac{1}{L_{ij}^{c}}+b$ with two unknown constants *a* and *b*. For simplicity, we assume that a=1 (or any constant we already know). Therefore, the reserve-ask is written as $A_{ij}^{c}=\frac{1}{L_{ij}^{c}}+b$. We now focus on estimating the constant *b*. If *b* is small and thus the reserve-ask is low compared to the supply, the slot may be sold at too low a price. On the contrary, if *b* is big and the reserve-ask is high compared to the supply, the channel slot may not be sold due to very limited supplies over the bar. Therefore, we guess that as *b* increases, the revenue of the PU increases first, reaches the maximum and then decreases. Therefore, we can simply model the revenue of the PU as the second order polynomial of *b* and try to find the optimal *b* that maximizes the revenue. It is is pertinent to mention that our choice of second order polynomial (or quadratic function) is guided by the shape of the relationships between the dependent constant, *b* and the explanatory variable (i.e., revenue of the PU) in the revenue model. We think that the shape of the relationship between dependent and explanatory variables is curved, so the second order polynomial is more suitable than the linear ones. A detailed study on quadratic function can be found in [63]. Precisely, we model:

(5) $$\begin{array}{cc} {\hat{R}}_{ij}^{c} & =\alpha b^{2}+\beta b+\gamma \\ & =\alpha {\left(A_{ij}^{c}-\frac{1}{L_{ij}^{c}}\right)}^{2}+\beta \left(A_{ij}^{c}-\frac{1}{L_{ij}^{c}}\right)+\gamma . \end{array}$$

The next step is to find the proper values of coefficients α, β, and γ for each period *n*. We know that at period *n*, we have n−1 previous data for ($L_{ij}^{c},A_{ij}^{c},{\hat{R}}_{ij}^{c}$). Therefore, we simply apply the least squares method to find the coefficients at each period. Given the coefficients at period *n*, our optimal *b* is $\frac{-\beta }{2\alpha }$, i.e., the reserve-ask is set as,

(6) $${\tilde{A}}_{ij}^{c}=\frac{1}{L_{i}^{c}}-\frac{\beta }{2\alpha }.$$

Hence, the ask of PU *j* in PS *i* can be defined as,

(7) $$A_{ij}^{c}={\tilde{A}}_{ij}^{c}.$$

#### 4.1.2. Bid Adaption

The SB naturally wants to obtain a large number of slots. To this end, SB can determine the optimal reserve-bid that maximizes its purchase. If the SB chooses the reserve-bid based on asks of the PSs, then SB can manipulate their bids to affect the auction results. Conversely, if SB chooses reserve-bid regardless of asks, it can fail to get the maximum number of slots with the good payments.

As a compromise, we can use the exponential weighted moving average model to figure out the projected value of the reserve-bid. We know that the exponential weighted moving average model decreases weight exponentially compared to that of the other time series weighted moving average models, which is what is more suitable for our targeted problem. This can indirectly provide a perspective for asks of spectrum slots in the future that is essential to forecast the sporadic shift in the secondary market. It is always better to use more recent information of spectrum slots, which is the better representative of what the future requires than the older one. That is, asks of channel slots in the last auction period are more relevant to asks for the next auction period. We put unequal weights on the past observations, that is larger weights on the least recent periods and comparatively smaller weights on the most recent periods. The exponentially-weighted moving average model for an auction period is defined as,

(8) $$\begin{array}{cc} {\hat{A}}^{c}= & \epsilon A_{n-1}^{c}+\left(1-\epsilon \right)A_{n-2}^{c}+{\left(1-\epsilon \right)}^{2}A_{n-3}^{c}\\ & \;\;\;+{\left(1-\epsilon \right)}^{3}A_{n-4}^{c}+\cdots +{\left(1-\epsilon \right)}^{\tau }A_{n-\tau }^{\tau }, \end{array}$$

where ${\hat{A}}^{k}$ refers to the SB’s projected ask of slots in channel *c* and auction period *n*. $A_{n-1}^{c}$, $A_{n-2,\cdots \tau }^{c}$ are the observed values of the auction period n−1, n−2,⋯τ, respectively. ϵ is the smoothing constant between zero and one (i.e., 0≤ϵ≤1). The least recent values of the asks are less responsive to the auction periods, meaning that they have very less significance over time. We therefore consider the information on τ terms, which are the known values of the past auction periods. Once the projected ask is known, we can find the reserve-bid, $B^{c}$ as,

(9) $${\tilde{B}}^{c}={\hat{A}}^{c}.$$

We use the value of smoothing constant, ϵ, between 0.2 and 0.3 for the low root means square error, as suggested in [64].

### 4.2. Auction Phase

In this phase, we discuss the design of inner- and outer-auctions. The key question is how to release the available slots in each channel while maintaining economic properties such as truthfulness, budget balance and individual rationality. This maps to the selection process of PUs in each PS to assemble the competing groups at inner-auction and the distribution of available slots to the SB at the outer-auction. Towards this end, we have designed the following auction mechanisms.

#### 4.2.1. Inner-Auction

The inner-auction takes place between the PUs and their serving PS. In Algorithm 1, we present the design of the inner-auction for PU *j*. In this algorithm, we select PUs with lower asks as the potential winner for available slots in each channel. That is, the selection of the winners is based on an asks-independent strategy, which is what is exclusively adopted by economist as in [13,15,36,39]. Otherwise, an ask-dependent selection can introduce a critical issue of ask manipulation, where participants driven by self-interest can strategically design asks to collude.
**Algorithm 1** Inner-auction algorithm in PS *i*.1:**for**c←1 to *C* **do**2: Let A be the ascending order array of asks $\left\{A_{ij}^{c}\right\}$;3: $\sigma_{i}^{c}\leftarrow N_{i}$-th element of A;4: $W_{i}\leftarrow \emptyset$;5: **for** j←1 to $N_{i}-1$ **do**6:  **if** $A_{ij}^{c}\leq \sigma_{i}^{c}$ & >0 **then**7:   $W_{i}\leftarrow W_{i}\cup \left\{j\right\}$;8:  **end if**9: **end for**10: $A_{i}^{c}\leftarrow k_{i}^{c}\times \sigma_{i}^{c}\times \left|W_{i}\right|$;11: $\left\{A_{i}^{c}\right\}\leftarrow A_{i}^{c}$;12:**end for**

According to Algorithm 1, PS *j* first sorts the received asks of PUs in ascending order. The PU with the highest ask is disqualified from the auction, which we call the sacrificed user. The ask information of the sacrificed user is used to define the clearing-price $\sigma_{ij}^{c}$ of slots in channel *c*. PS *j* then chooses the PUs with asks less than the clearing price as potential winners (Lines 2–7). Finally, we set the group asks $A_{i}^{c}$ of channel *c* as,

(10) $$A_{i}^{c}=k_{i}^{c}\times \sigma_{i}^{c}\times \left|W_{i}\right|.$$

Later on, PS *j* submits group-ask, $A_{i}^{c}$, to the outer-auction for each channel. We need to mention here that the result of the inner-auction is announced to the potential winners once their serving PS has won rights to sell group-slots in the outer-auction.

**Figure 5:**
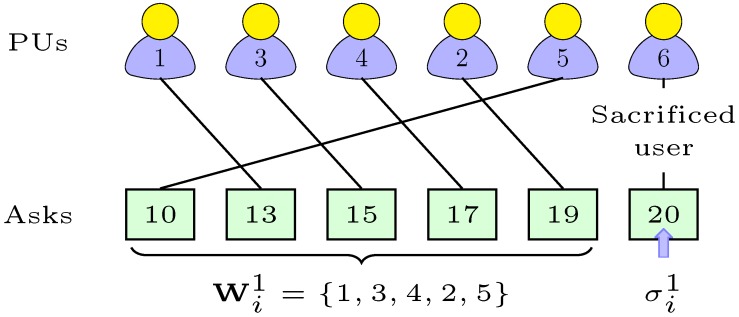
Illustration of the inner-auction algorithm.

Example: Let us illustrate our inner-auction algorithm with the help of a toy example. In our example, there are six PUs (or sellers) in the auction process with one slot each in Channel 1, as shown in Figure 5. Therein, each solid line connects the true bid and the same associated seller. First, the serving PS sorts asks in ascending order, chooses the highest ask, i.e., 20, as the clearing price and drops Seller 6 as the sacrificed user. The serving PS then finds that asks of the remaining five PUs qualify for the clearing price, so it chooses them as the potential winners and sets the group-ask $A_{i}^{1}=100(=1\times 20\times 5$). Now, if the serving PS wins the right to sell in the outer-auction, then each winner PU will receive 20 units of money per each slot in Channel 1. Unlike the ask-independent strategy, if the selection of the winners is based on their own asks, then selfish sellers could submit the fake asks. Refer to the same example: if all the winners keep their submissions truthful except the Winner 1, who submits the fake ask of 19 instead of 10, it will still remain the winner, but is liable to receive the same amount of money, i.e., 20, and any further increase in ask value will make it a loser. Therefore, it has obviously no incentive to do so.

#### 4.2.2. Outer-Auction

We now discuss the outer-auction, which is carried out between PSs and SB. In Algorithm 2, each PSs can at most sell slots of one channel. That is, once a PS sells the slots of a channel, it will not be considered for any further selling in the same auction period. Moreover, slots in all the channels are sold at random to avoid a collision. We hence denote the random set of available slots in all the channels as C.
**Algorithm 2** Outer-auction algorithm in SB.1:Let M be the ask-matrix {$A_{i}^{c}$} for all *i* and *c*2:Let C be the random set of all the channels’ slots3:Let N be the set of qualified primary sellers4:**for**c←1 to |C| **do**5: W←∅;6: **while** $A_{i}^{c}\leq {\tilde{B}}^{c}$ & >0 for all i∈M **do**7:  $N\leftarrow A_{i}^{c}$;8:  Sort N in descending order;9:  **if** |N|>1 **then**10:   Choose first element $e_{1}\in N$;11:   Choose an element $e_{r}\left(<e_{1}\right)\in N$ randomly;12:  **else**13:   break;14:  **end if**15:  $W\leftarrow W\cup \{e_{r}\}$;16:  $P^{c}=\left\{e_{1}\right\}$;17:  $M=M\backslash \{e_{r}$’s asks}; C=C\{c};18: **end while**19:**end for**

### 4.3. Remuneration Phase

According to Algorithm 2, SB receives the asks from PSs on slots in all the channels and finalizes its reserve-bid-matrix, i.e., $\left\{B^{c}\right\}$ from (9). It sorts the received asks $\left\{A_{i}^{c}\right\}$ for all *i* and *c* in an ask-matrix denoted as M. For each channel, SB evaluates all the elements of $\left\{A_{i}^{c}\right\}$ compared to each element of $\left\{B^{c}\right\}$ and constructs a set of qualified PSs denoted as N. A PS can be chosen as the qualified seller if its ask $A_{i}^{c}$ is less than or equal to the reserve-bid ${\tilde{B}}^{c}$. Otherwise, it is dropped from the contest of that channel (Lines 5 to 7). Then, SB sorts the N set in descending order and chooses the topmost element $e_{1}$ provided the qualified sellers are more than one. If that is not the case, no PS can sell the slots on that channel. SB drops the PS of this topmost element due to the highest ask. From the reaming eligible sellers, SB chooses another element $e_{r}$ at random such that it should be less than $e_{1}$ (Lines 8 to 13). The chosen elements $e_{r}$ and $e_{1}$ define the winning PS and price of that channel, respectively (Lines 15 to 16). Finally, the winning seller (who reserves the right to sell slots in the channel) and the sold channel are removed from the ask-matrix and channel set, respectively (Line 17). The PS *j* ∈W will reap the revenue of:

(11) $$R_{i}=P^{c},$$

when it has sold slots in the channel *c*.

**Figure 6:**
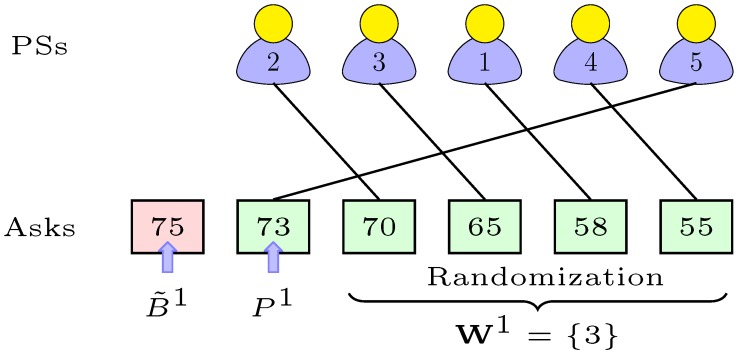
Illustration of the outer-auction algorithm.

Example: Let us consider an example of the outer-auction algorithm in Figure 6. In our example, each solid line connects the true ask to the same associated PS. For channel *c*, we assume five PSs in the contest and reserve-bid ${\tilde{B}}^{c}=75$. The set of eligible PSs is $W^{1}=\left\{1,2,3,4,5\right\}$. In that case, PS 5 becomes dropped due to the highest ask, which is $e_{1}\left(=73\right)$. Suppose that PS 3 is randomly chosen out of the remaining four PSs whose asks are less than $e_{1}$. Then, PS 3 becomes the winner, and the sold price of slots in Channel 1 is set to 73.

In this phase, we discuss what payment of the channel slots is made to the winning PUs in the inner-auction. We know that the winner set $W_{i}^{c}$ of the inner-auction has been already constructed for each channel *c*. Thus, the winner set $W_{i}^{c}$ is notified to the corresponding PUs when PS i∈W receives notification from the SB as the winning seller of slots in channel *c*.

The PS *i* gets revenue from the SB first and then pays to the winning PUs for the slots sold in channel *c*. In AERG, the PS *i* exactly pays the amount of:

(12) $$R_{ij}=\frac{A_{i}^{c}}{|W_{i}|}\times k_{i}^{c}=\sigma_{i}^{c}\times k_{i}^{c}$$

Furthermore, PUs can reap revenue proportional to the number of sold slots, so that they are motivated to release more and more spectrum. It can be a straightforward approach to adopt a discriminatory mechanism such that PU $j\in W_{i}^{c}$ receives revenue proportional to its ask. However, in that case, PUs are motivated to increase their revenue with the fake asks.

## 5. Properties of AERG

We now prove that our proposed AERG framework holds the good properties of an economic-robust auction design. If the auctioneer of an auction gets non-negative utility, then it can be called budget-balanced. We here prove that in our proposed framework, the auctioneers of inner-auction and outer-auction both can earn the non-negative utility.

**Theorem** **1.** AERG is budget-balanced.

**Proof.**  (1) Inner-auction: We know that the auctioneer of the inner-auction is each PS. If a PS *i* fails to sell slots to SB, then its utility will be equal to zero. Hence, we assume that PS has sold spectrum slots to the SB. Then, its utility can be written as $U_{i}=R_{i}-\sum_{j\in W_{i}}R_{ij}=P^{c}-A_{i}^{c}$ since $R_{ij}=\frac{A_{i}^{c}}{|W_{i}|}\times k_{i}^{c}$ from Equation (12) and $R_{i}=P^{c}$ from Equation (11). According to Algorithm 2, SB chooses the PS 1 and PS *r* from the eligible candidates, then the highest ask of PS 1 is greater than or equal to that of PS *r* (lines 10–11). That is, $A_{1}^{c}\geq A_{r}^{c}$ or $\left(P^{c}=R_{i}\geq A_{i}^{c}\right)$. In that cases, $U_{i}=R_{i}-A_{i}^{c}\geq 0$.

(2) Outer-auction: We recall that the auctioneer of the outer-auction is the SB, whose utility can be written as $U={\tilde{B}}^{c}-\sum_{c}P^{c}$ from (3). From Algorithm 2, the reserve-bid $B^{c}$ (or SB’s evaluation) on each channel *c* is larger than or equal to $A_{i}^{k}$ (Line 6). Hence, the utility $U={\tilde{B}}_{i}^{c}-A_{i}^{c}\geq 0$. ☐

We now discuss the next economic property of our AERG framework. A reverse-auction is individually rational if the winning seller has paid more than its submitted ask.

**Theorem** **2.** AERG is individually rational.

**Proof.**  (1) Inner-auction: We know that the seller of the inner-auction is each PU. We assume that PU *j* in PS *i* has sold his $k_{i}^{c}$ slots to the SB. According to Algorithm 1, PU *j* has submitted the ask ${\hat{A}}_{ij}^{c}\leq \sigma_{i}^{c}$ (Line 6), and gets revenue $R_{ij}=\sigma_{i}^{c}\times k_{i}^{c}$ from Equation (12). Therefore, ${\hat{A}}_{ij}^{c}\leq R_{ij}$.

(2) Outer-auction: We know that the seller of the outer-auction is each PS. We assume that PS *i* has sold $k_{i}^{c}$ slots in channel *c*, it has submitted the ask $A_{i}^{c}$ and obtained revenue $R_{i}=P^{c}$ as from (11). Since $A_{i}^{c}\leq P^{c}$ (refer to the proof of Proposition 1), $R^{c}\geq A_{i}^{c}$. Hence, it is guaranteed that each PS is paid more than its submitted ask. ☐

We now recall the next property of the AERG framework. A reverse-auction is truthful (or strategy proofed) if a seller cannot improve its utility by submitting the fake ask.

**Theorem** **3.** AERG is truthful.

**Proof.**  (1) Inner-auction: We know that our Algorithm 1 works on an ask-independent strategy. Therein, an PU cannot improve its utility by a fake ask in the following senses.

(i) PU *j* is the looser: The utility of the looser (or sacrificed user) is zero since it cannot sell slots to the secondary market. We henceforth denote the fake asks with a unique symbol as $\vec{A}$. If the PU submits a fake ask ${\vec{A}}_{ij}^{c}$ smaller than or equal to the clearing price, i.e., ${\vec{A}}_{ij}^{c}<\sigma_{i}^{c}$, it can be selected as the seller of $k_{i}^{c}$ slots. However, it is paid then $R_{ij}\leq {\tilde{A}}_{ij}^{c}$ from Equation (12). Therefore, the utility of PU *j* becomes $U_{ij}=R_{ij}^{c}-{\tilde{A}}_{ij}^{c}\leq 0$, and it has no incentive to do so.
(ii) PU *j* is the winner: The PU *j* becomes the winner seller of $k_{i}^{c}$ slots, and its maximum revenue is $R_{ij}$. The utility of PU *j* is given as $U_{i}-R_{ij}-{\tilde{A}}_{ij}^{c}$. Note that $R_{ij}$ remains unchanged, although PU *j* submits the fake ask because the clearing price is independent. Hence, the PU *i* cannot improve its utility with the fake ask.

(2) Outer-auction: The seller of the outer-auction is each PS *i*. In this connection, we have shown the following three cases.

(i) PS *i* is not qualified: The utility of PS *i* is zero because it cannot sell the spectrum slots. Suppose that the PS submits a fake ask ${\vec{A}}_{i}^{c}$ smaller than or equal to the reserve-bid, i.e., ${\vec{A}}_{i}^{c}\leq B^{c}$, it can be selected as the winner. However, PS then gets payment $R_{i}\leq A_{i}^{c}$, and its utility becomes $U_{i}=R_{i}-A_{i}^{c}\leq 0$. On the other hand, it cannot control the reserve-bid ${\tilde{B}}^{c}$ since it is independent of the PS *i*’s ask $A_{i}^{c}$ and the section of the winner is purely a random process. Therefore, it is guaranteed that no PS can increase its utility with the untruthful ask.
(ii) PS *i* is qualified, but not the winner: The utility of PS *i* is zero because it is a loser. If PS *i* untruthfully submits the lower ask to become a winner, but the gap between its highest ask and the randomly chosen ask is unknown, it remains a loser. Even if PS submits a fake ask ${\vec{A}}_{i}^{c}$ smaller than or equal to the reserve-bid, i.e., ${\vec{A}}_{i}^{c}\leq B^{c}$, it is selected as the winner. However, it is paid then $R_{i}\leq A_{i}^{c}$. In that case, the utility becomes $U_{i}=R_{i}-A_{i}^{c}\leq 0$. Therefore, PS *i* cannot increase its utility with the fake ask.
(iii) PS *i* is the winner: Denoting the other qualified seller as PS 2, the utility of PS *i* is written as $U_{i}=A_{2}^{c}-A_{i}^{c}\geq 0$. Suppose that PS *i* submits the fake ask ${\vec{A}}_{i}^{c}>A_{2}^{c}$; it can increase the price of the spectrum slots to ${\vec{P}}_{i}^{c}>P_{i}^{c}$. However, it cannot be a winner anymore due to the highest ask, and its utility becomes zero. Hence, PS *i* cannot increase its utility by the untruthful ask.

 ☐

## 6. Performance Analysis

In this section, we evaluate the performance of our AERG framework in terms of agents’ utilities and social welfare, as defined in Equations (1)–(4) of the system model. We also verify that our proposed system bears good qualities such as adaptive behavior and the economically-robust mechanism. We compared the results of the AERG system with that of its non-adaptive mechanism called Static and Economically-Robust Group selling (SERG) to investigate the performance.

### 6.1. Simulation Setup

We have developed the simulation code in MATLAB, which follows all the details of our AERG framework as described in Section 4. We have set the default parameters i.e., number of PSs, N=5 and number of PUs, $N_{i}$ = 15, for the *i*-th primary network. We assume each PS *i* has one channel in its serving network and each PU *j* can release at least one slot in that channel. For slots in channel *c*, asks $A_{ij}^{c}$ of PU *i* in PS *j* are uniformly distributed in [2,6]. In AERG, SB adapts reserve-bids ${\tilde{B}}^{c}$ from the PSs’ ask of the past 100 periods. However, SB in SERG selects bids in [55,75] with a uniform distribution due to the non-adaptive mechanism. We also need to mention that all simulation results are the average of 100 runs.

### 6.2. Results’ Discussion

**Figure 7:**
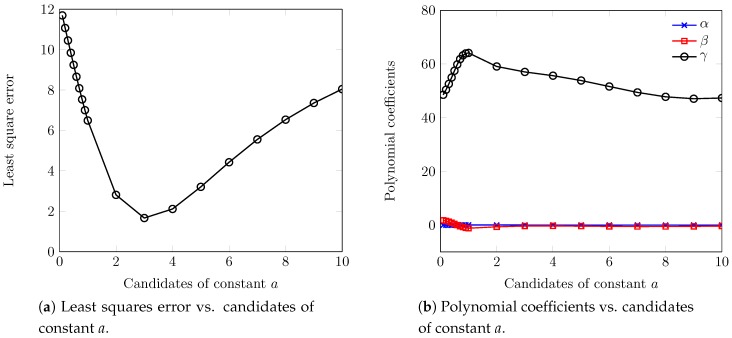
Lest squares error and polynomial coefficients at various candidates of constant *a*.

Figure 7a demonstrates the least squares errors at different values of constant *a*. We observe that constant a=3 has the lowest error, which is used to figure out the optimal value of constant *b*. From the optimal values of constants *a* and *b*, we then derive the optimal values of the coefficients (α, β, γ) (see Figure 7b) to adapt the PU *i*’s ask at the inner-auction.

**Figure 8:**
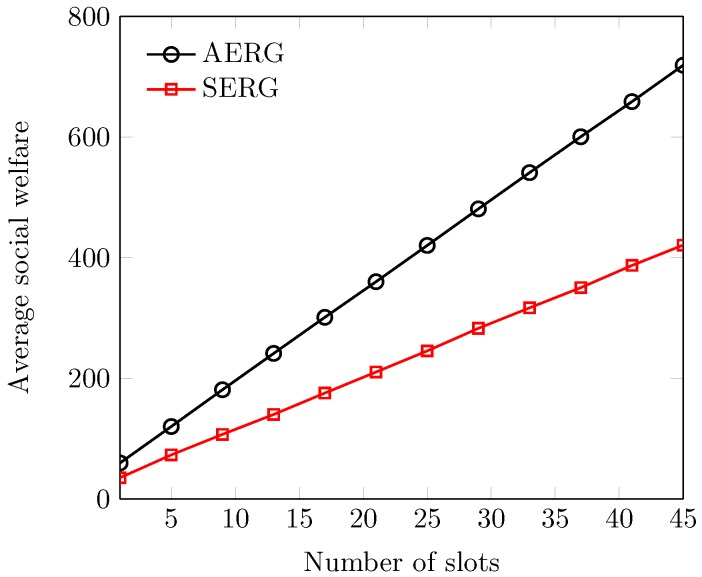
Social welfare vs. number of slots. SERG, Static and Economically-Robust Group selling.

**Figure 9:**
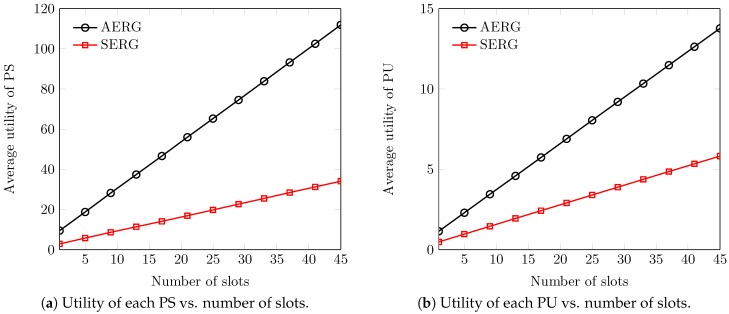

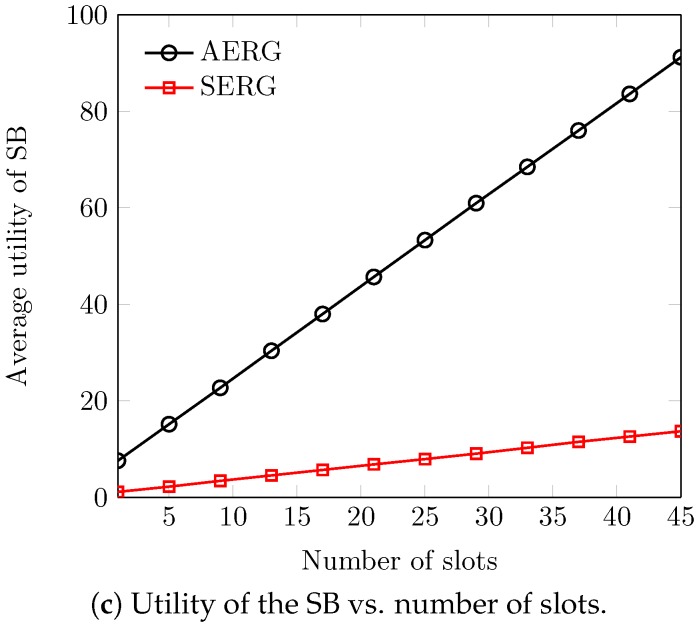
Average utilities of the agents vs. number of the slots.

In Figure 8, we have presented the average results of social welfare for the various number of spectrum slots. We witness that social welfare is a function of slots sold in the secondary market. This is simply because with more spectrum (or slots), there is more performance gain in social welfare. However, the gap between the curves of social welfare is attributed to the adaptive and random evaluation of slots by AERG and SERG, respectively. The slot selling ability of AERG increases due to fair evaluation and so increases the social welfare. Figure 9 describes the average utilities of the agents at different numbers of slots. We observe that the average utility of each participant increases with the increase in the number of slots. This is good evidence because, with more and more released slots, the utilities of the participants remain high due to the economies of large-scale selling. In this connection, we also observe that AERG outperforms SERG in both inner- and outer-auctions. This is because of the fact that AERG enables the buyer and sellers to evaluate the spectrum slots fairly considering the dynamics of time, space and availability. More precisely, each PU *i* adapts its ask from the slots’ availability in each channel for each period for the outer-auction. Similarly, the SB adapts its reserve-bid from each PS *j*’s asks of past τ terms. This simply enables both buyers and sellers to learn each other’s acceptable evaluation without compromising the truthfulness of the system. However, the agents in SERG cannot sell more spectrum due to their blind valuations that eventually lead to a decrease of their utilities.

**Figure 10:**
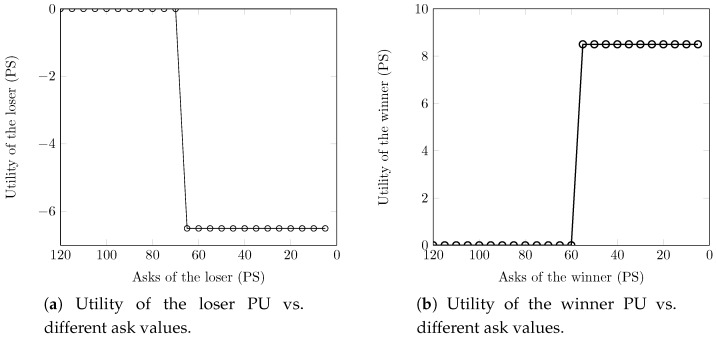

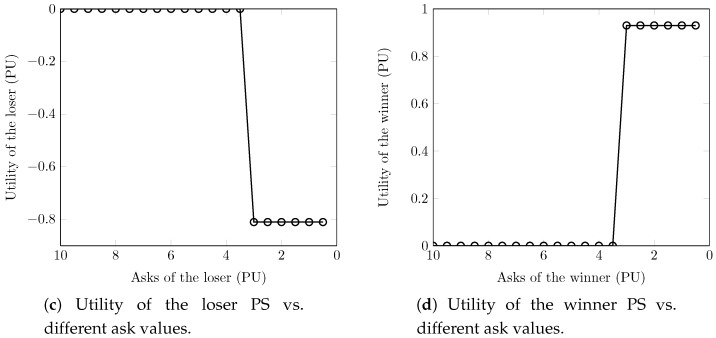
Truthfulness of AERG for the inner-/outer-auction.

We here demonstrate the truthfulness of AERG at the inner- and outer-auctions. We recall that sellers of the inner- and outer-auctions are PUs and PSs, respectively. A seller cannot sell its slots without participating in the auction, and in that case, its utility will be equal to zero. Furthermore, the loser (PU/PS) will have zero utility. Figure 10 shows the truthfulness of our AERG scheme. We randomly choose one loser and one winner in order to investigate how their utilities affect when they submit fake asks at the inner-auction. As shown in Figure 10a, the loser PU gets zero utility with the true ask of 5.3. We witness that the utility of the loser becomes negative if the PU submits a fake ask smaller than 5.3, and it remains a loser with asks larger than 5.3. On the other hand, the winner PU obtains positive utility with the true ask of three, as shown in Figure 10b. We observe that winner becomes a loser and obtains zero utility if PS submits a fake ask larger than three. Moreover, the winner remains a winner if the PS submits a fake ask smaller than the true ask. In the outer-auction, we also pick one loser and one winner to investigate how their utilities affect when submitting the fake asks. The loser PS obtains zero utility with the true ask of 67, and it gets negative utility with fake asks smaller than the true ask, as shown in Figure 10c. Further, the PS loser’s fake asks larger than the true ask have no effect anyway. On the contrary, the winner gets positive utility with the true ask of 55, as shown in Figure 10d. The winner PS becomes the loser with the fake asks are larger than 55. Further, an increase in ask does not affect the winner’s utility.

## 7. Conclusions

Spectrum is an increasingly scarce resource that needs to be efficiently managed if more cannot be created. Traditional spectrum auctions enable measurable return, but are not essentially efficient, because distributing channels only at the macro level renders the spectrum scarce. However, the channel distribution at the micro level is facing various challenges in the secondary market such as spectrum heterogeneity, lack of legislation, interference management, and so on. We can avoid spectrum scarcity by protecting the incentives of the micro-level spectrum holders, like PUs in CR networks. If a PU has a limited number of slots, buyers are then least interested in purchasing those slots because they are not sufficient for their needs. To this end, a buyer has to deal with multiple sellers, which can not only increase the auction overhead, but can also hold back its interest. Moreover, the true evaluation of the slots is immensely important. However, the evaluation of non-identical channels is very difficult, as the price paid for one specific channel may not be a useful indicator of the valuation of another channel. Ultimately, the spectrum holders may fail to set reasonable asks, which can affect the auction results and then the spectrum efficiency. In the secondary market, the group-selling and participants’ fair valuations of idle slots are of great interest. In this connection, we have proposed AERG, which is the first group-selling mechanism of spectrum slots for the PUs in the secondary market. Therein, PUs in the same channel can fairly evaluate and aggregate their small number of slots at one time and space in one group to jointly trade them in an economically-robust environment. In group-selling, it should be noticed that the revenue of the PUs is increased since more spectrum is released due to the increased market value of aggregated slots. The SUs can enjoy more spectrum of choice, which is otherwise not used, to contribute towards spectrum efficiency and social welfare. Simulation results verify that AERG is the best candidate mechanism of scarce spectrum trading at the micro level.

## Figures and Tables

**Table 1:** Classification of proposed AERG framework and other related schemes.

	Category	**Individual-Buying**	**Group-Buying**	**Group-Selling**
Auctions				
WERITAS		✓	✗	✗
SMALL		✓	✗	✗
TRUST		✓	✗	✗
TASC		✓	✗	✗
TAHES		✓	✗	✗
SMASHER		✓	✗	✗
TASG		✗	✓	✗
DEAL		✗	✓	✗
TRUBA		✗	✓	✗
AERG		✗	✗	✓

**Table 2:** Summary of notations.

Notation	Description
*N*	Number of PUs
$N_{i}$	Number of PUs served by PS *i*
*C*	Number of available channels
$k_{i}^{c}$	Number of free uplink slots in channel *c* at PS *i*
${\hat{A}}^{c}$	SB’s projection of PS *i*’s ask on slots in channel *c*
${\hat{R}}_{ij}^{c}$	PU *j*’s projected revenue on slots in channel *c* in PS *i*
$A_{i}^{c}$	PS *i*’s ask on $k_{i}^{c}$ slots in channel *c*
${\tilde{B}}^{c}$	SB’s reserve-bid of slots in channel *c*
$A_{ij}^{c}$	PU *j*’s ask on slots in channel *c* (at PS *i*)
${\tilde{A}}_{ij}^{c}$	PU *j*’s reserve-ask of slots in channel *c*
$L_{ij}^{c}$	Availability of slots in channel *c* at PU *j*
$P^{c}$	Slots price of channel *c*
W	Winner set of the outer-auction
$W_{i}$	Winner set of the inner-auction at PS *i*
$R_{i}$	Revenue of PS *i* from the SB
$R_{ij}$	Revenue of PU *j* from PS *i*
*U*	Utility of the SB
$U_{i}$	Utility of the PS *i*
$U_{ij}$	Utility of the PU *j* located in PS *i*
*S*	Social welfare
